# Loss of the SIN3 transcriptional corepressor results in aberrant mitochondrial function

**DOI:** 10.1186/1471-2091-11-26

**Published:** 2010-07-09

**Authors:** Valerie L Barnes, Bethany S Strunk, Icksoo Lee, Maik Hüttemann, Lori A Pile

**Affiliations:** 1 Department of Biological Sciences, Wayne State University, 5047 Gullen Mall, Detroit, Michigan 48202, USA; 2 Center for Molecular Medicine and Genetics, Wayne State University School of Medicine, 540 E. Canfield, Detroit, Michigan 48201, USA; 3 Chemical Biology, University of Michigan, 930 N. University Ave. Rm. 4250, Ann Arbor, Michigan 48109, USA

## Abstract

**Background:**

SIN3 is a transcriptional repressor protein known to regulate many genes, including a number of those that encode mitochondrial components.

**Results:**

By monitoring RNA levels, we find that loss of SIN3 in *Drosophila *cultured cells results in up-regulation of not only nuclear encoded mitochondrial genes, but also those encoded by the mitochondrial genome. The up-regulation of gene expression is accompanied by a perturbation in ATP levels in SIN3-deficient cells, suggesting that the changes in mitochondrial gene expression result in altered mitochondrial activity. In support of the hypothesis that SIN3 is necessary for normal mitochondrial function, yeast *sin3 *null mutants exhibit very poor growth on non-fermentable carbon sources and show lower levels of ATP and reduced respiration rates.

**Conclusions:**

The findings that both yeast and *Drosophila *SIN3 affect mitochondrial activity suggest an evolutionarily conserved role for SIN3 in the control of cellular energy production.

## Background

Mitochondria are dynamic organelles whose function and total number must be responsive to the energy demand of the cell. When cellular energy demand is high, expression of genes encoding mitochondrial factors must be up-regulated. Although the mitochondrial genome encodes components of the oxidative phosphorylation pathway, the majority of the proteins required for mitochondrial biogenesis and function are encoded by the nuclear genome. While a number of transcription factors, including NRF1, NRF2, PPARα and PPARγ, are required for activation of many nuclear encoded mitochondrial genes, little has been reported regarding repression of these same genes [1].

Mitochondria contain three important classes of proteins encoded by nuclear genes [2]. The first class includes the catalytic and auxiliary proteins of the numerous enzyme systems. These include enzyme systems involved in fatty acid oxidation, the citric acid cycle, oxidative phosphorylation, and the removal of reactive oxygen species (ROS). A second class of mitochondrial proteins includes components required for protein import of mitochondrial factors into the mitochondrial intermembrane space, the inner membrane, and the mitochondrial matrix. The third class is comprised of the enzymes and additional proteins required for replication and translation of the mitochondrial genome which are thus required for mitochondrial biogenesis. Subsets of mitochondrial genes have been shown to increase or decrease in expression under a variety of tested conditions, suggesting that these groups of genes are co-regulated [3,4]. We previously determined that many nuclear encoded mitochondrial genes are subject to regulation by the SIN3 transcriptional corepressor [5]. Elimination of SIN3 in *Drosophila *S2 tissue culture cells by RNA interference (RNAi) led to an increase in the expression of a substantial number of genes encoding mitochondrial proteins in all three classes [5]. The SIN3-deficient cells were also found to have an increase in mitochondrial mass.

Although *Drosophila *SIN3 is required for viability during both embryonic and larval stages of development, the exact nature of the essential requirement for SIN3 is not understood [6-8]. SIN3 is a component of the SIN3 histone deacetylase complex, required for general repression of transcription [9]. RPD3 is the catalytic component of the SIN3 complex [10-12]. Similar to SIN3, loss of *Drosophila *RPD3 leads to loss of viability as well as alterations in mitochondrial function and gene activity [13-16]. Loss of either SIN3 or RPD3 causes an increase in mitochondrial citrate synthase activity, indicating that mitochondrial oxidative capacity is affected following decreased expression of key SIN3 complex components [13].

Given the finding that the SIN3 corepressor affects expression of nuclear genes encoding mitochondrial proteins, we decided to further investigate the relationship between SIN3 and mitochondrial gene activity and function. We find that similar to the increase in expression of the nuclear encoded mitochondrial genes, we see an increase in expression of genes encoded by the mitochondrial genome. This elevation is due to increased transcription as the number of mitochondrial DNA genomes is unchanged in the SIN3-deficient cells. Additionally, we find that the increased expression of genes encoding proteins with mitochondrial function is accompanied by an alteration in ATP levels. These results suggest that the changes in gene expression give rise to altered mitochondrial function. In support of these findings yeast *sin3 *null mutants grow very poorly on media containing only non-fermentable carbon sources. The *sin3 *null strain also exhibits lowered levels of ATP and respiration rates relative to wild type. The data implicate SIN3 as having a role in the control of cellular energy production.

## Results

### SIN3 regulates genes involved in mitochondrial physiology

**Figure 1:**
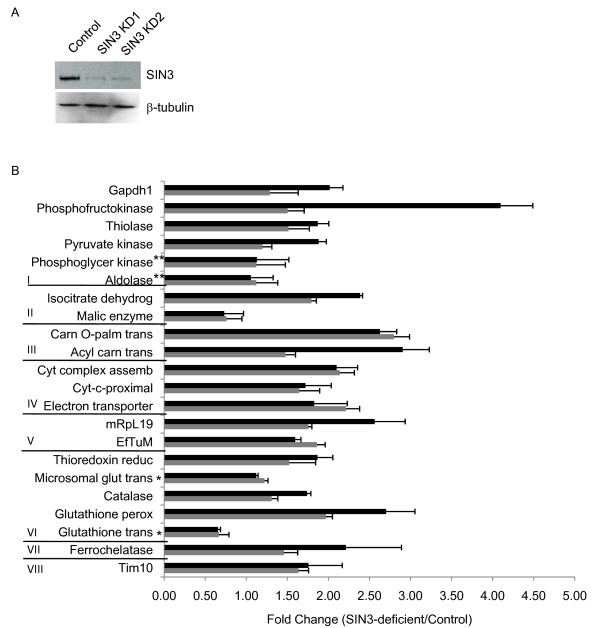
**Expression of nuclear encoded genes involved in multiple mitochondrial processes is up-regulated following loss of SIN3**. SIN3-deficient cells were generated by RNAi methodology using dsRNA generated against different regions of the SIN3 transcript KD1 (black bars) or KD2 (gray bars). (A) Western blot analysis of whole cell extracts prepared from control or SIN3-deficient cells as indicated. The blots were probed with antibody to SIN3 or to β-tubulin as a loading control. (B) qPCR was performed using cDNA prepared from control and SIN3-deficient cells as a template and primer pairs for the genes indicated along the Y-axis. Relative levels of gene expression are indicated. *Genes with decreased expression in microarray. **Genes with no expression level change in microarray. Genes are grouped according to process: I-glycolysis, II-citric acid cycle, III-fatty acid oxidation, IV-oxidative phosphorylation, V-mitochondrial biogenesis, VI-reactive oxygen species (ROS) pathways, VII-heme synthesis, and VIII-transporter. Error bars represent standard error (n = 3-6 for KD1 and n = 3-5 for KD2). p ≤ 0.05 for all data.

Previously published research from one of the authors (LAP) revealed that reduction of SIN3 by RNAi results in up-regulation of numerous genes involved in multiple aspects of mitochondrial biology, including activity and biogenesis [5]. Using RNAi methodology in cultured *Drosophila *cells, we have a tractable system that enables us to induce a group of co-regulated genes by loss of the corepressor SIN3. This system allows us to monitor and compare molecular events that occur before and after induction of the different classes of mitochondrial genes including those of the enzyme systems, protein import, and biogenesis. Because SIN3 regulates many genes, we selected a representative set for analysis (Figure 1B, Additional file 1). Genes encoding each mitochondrial protein class are represented in the up-regulated genes. We chose at least one gene from each class. A number of genes encoding proteins involved in the numerous enzyme systems were selected. We are thus able to compare the regulation of genes within and between distinct mitochondrial processes. We chose genes encoding proteins of the individual energy pathways of the enzyme systems including fatty acid oxidation, citric acid cycle, oxidative phosphorylation, and the removal of ROS. Expression of genes encoding proteins involved in glycolysis, which takes place in the cytoplasm, but produces pyruvate used in mitochondria were also analyzed. A number of the selected genes are known to be regulated by trans-factors that have a putative link to either SIN3 or histone deacetylation. For example, mRpL19, EfTuM, ferrochelatase, and Tim10 were all shown to be bound by one or more members of the Max family network of transcription factors [17]. Repression by the Max family member Mad is mediated through interactions with SIN3 [18]. Gapdh1 and pyruvate kinase are glycolytic genes regulated in part by HIF-1 [19]. HIF-1 associates with FIH-1 which mediates repression of HIF-1 through interaction with HDAC-1 [20]. Cytochrome *c *(*Drosophila *CG2140) and PHGPx are regulated in part by Sp1 that has been shown to interact with SIN3 [21-23]. Thiolase and acyl carnitine transporter (*Drosophila *CG3476) have been found to be regulated by PPARα [24,25]. Multiple PPARα-regulated genes are induced by treatment with histone deacetylase inhibitors [26]. Thioredoxin reductase is also activated following inhibition of histone deacetylation [27].

To extend the previously published microarray data and to determine the relative difference in expression, we analyzed gene expression by quantitative RT-PCR analysis. To ensure that the changes in gene expression were due to loss of SIN3 and not the result of an off target effect [28], we generated double stranded RNA (dsRNA) against two different regions of the SIN3 gene. The amount of SIN3 knockdown was monitored by Western blot analysis of whole protein extracts prepared from the cells. SIN3 expression was reduced to similar levels in cells incubated with dsRNA targeting the two different sequences of the SIN3 mRNA (Figure 1A). cDNA made from RNA isolated from each of the cell populations, control, SIN3-deficient 1 (KD1), and SIN3-deficient 2 (KD2), was analyzed by a quantitative real-time PCR (qPCR) assay. The fold change in expression of the selected genes is given in Figure 1B. The majority of the genes selected (Additional file 1) were previously identified as SIN3 targets by microarray analysis of RNAi-induced SIN3-deficient cells [5]. We also included two genes, phosphoglycerate kinase and aldolase, which did not show a change in expression by the microarray analysis, as well as two genes, microsomal glutathione transferase 1 (Mgst1) and CG11784 (glutathione transferase), that showed down-regulation in the microarray study. The increases in gene expression following loss of SIN3 using the same dsRNA (KD1) as in the microrarray experiment ranged from 1.6 to 4.1 fold, consistent with the microarray data [5]. Use of dsRNA targeting a distinct sequence of SIN3 (KD2) also resulted in up-regulation of the majority of genes tested, although for some of these, the amount of increase was less than 1.5 fold. CG11784 (glutathione transferase), one of the two down-regulated genes tested, showed a decrease following SIN3 knockdown using either of the dsRNAs. The other gene, Mgst1, that by microarray was found to be down-regulated, showed essentially no change in expression as measured by qPCR. The phosphoglycerate kinase and aldolase genes had similar levels of expression in the control and SIN3-deficient cells, consistent with the microarray data. The results of this qPCR experiment confirm our previous findings that loss of SIN3 results in the coordinated up-regulation of genes encoding proteins involved in multiple mitochondrial functions. We saw similar patterns of changes in gene expression with either of the SIN3 targeting dsRNAs, indicating that the observed effects are likely due to loss of SIN3 and are not the result of an off target effect.

### Mitochondrial encoded genes are up-regulated following loss of SIN3

**Figure 2:**
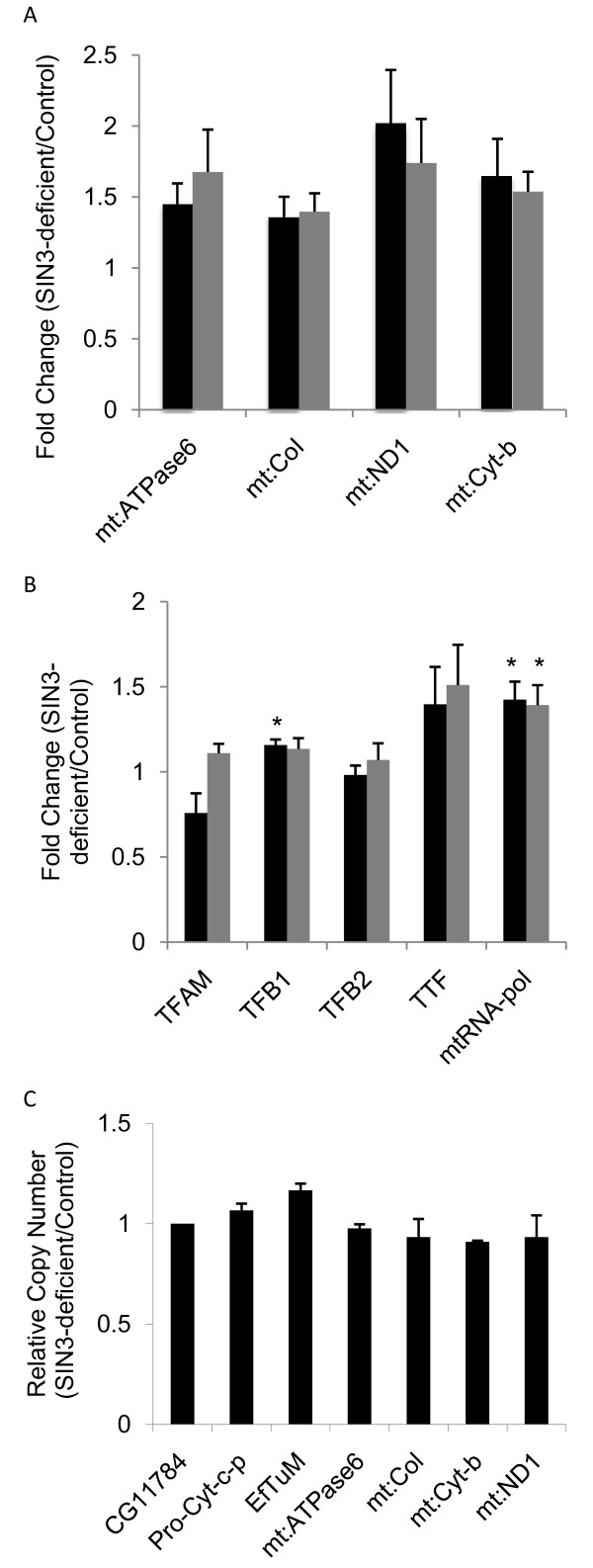
**Analysis of mitochondrial gene expression and genome copy number**. Expression analysis of mitochondrial encoded genes (A) and nuclear encoded mitochondrial transcription factors (B). Total RNA isolated from control and SIN3-deficient cells (KD1, black bars, and KD2, gray bars) was used to generate cDNA. qPCR was performed using the cDNA as a template and primer pairs for the genes indicated along the X-axis. Relative levels of gene expression are indicated. Error bars represent standard error. A, n = 7 for KD1 and n = 5 for KD2, p < 0.05 for all data. B, n = 4, *p < 0.05. (C) Mitochondrial DNA copy number is unaltered following loss of SIN3. DNA was isolated from control and SIN3-deficient cells. qPCR was performed using the DNA as a template and primer pairs for both nuclear and mitochondrial genome sequences as indicated along the X-axis. Pro-cyt-c-p amplifies the promoter region of Cyt-c-p. Relative copy number is indicated. The nuclear gene glutathione transferase (CG11784) was used for normalization. Error bars represent standard error (n = 3).

Because loss of SIN3 resulted in increased expression of nuclear encoded mitochondrial genes, we next examined expression of genes encoded by the mitochondrial genome in SIN3-deficient cells. We assayed RNA levels using qPCR analysis with primers designed to individually amplify four distinct mitochondrial encoded genes. cDNA made from RNA isolated from control and SIN3-deficient S2 cells was used as template for qPCR. Loss of SIN3 resulted in increased expression of the four mitochondrial genes tested, mt: ATPase6, mt: CoI, mt: Cyt-b and mt: ND1 (Figure 2A). Interestingly, the increase in expression of the mitochondrial encoded genes in the SIN3-deficient cells was very similar to the level of increase in expression of the nuclear encoded genes. The changes were in the range of 1.4 to 2.0 fold. Mitochondrial encoded genes are regulated by transcription factors that are encoded by the nuclear genome. We determined whether nuclear encoded transcription factors, including TFAM, TFB1, TFB2, mitochondrial RNA polymerase (mtRNA-pol) and transcription termination factor DmTTF, showed a change in expression following loss of SIN3. While the majority of the factors did not show a significant increase in expression in the SIN3-deficient cells, mtRNA-pol was up-regulated 1.4 fold relative to control cells (Figure 2B). Taken together, the data suggest that the increase in the levels of the tested mitochondrial transcripts is possibly due to an increase in the level of mtRNA-pol. The finding that the increases in expression of the nuclear and mitochondrial encoded genes are all in the same range is consistent with numerous reports outlining cross-talk between the nuclear and mitochondrial genomes [29].

The up-regulation of mitochondrial ribosomal and translation elongation proteins suggests that mitochondrial biogenesis is increased in SIN3-deficient cells. In addition, SIN3-deficient cells have more mitochondrial mass compared to control cells [5]. Given these findings, we decided to determine the relative mitochondrial DNA copy number in control and SIN3-deficient cells. DNA extracted from each of the samples was subjected to qPCR analysis using primer pairs specific for mitochondrial DNA. As a control, we also determined the relative levels of two nuclear encoded genes. Based on relative levels of four mitochondrial genes, we found that mitochondrial DNA copy number was unchanged following loss of SIN3 (Figure 2C). The finding that the transcripts of these mitochondrial encoded genes are increased while mitochondrial genome copy number is unchanged suggests that, in SIN3-deficient cells, there is an increase in the amount of mitochondrial gene transcription from the equivalent number of genomes rather than similar levels of transcription from an increased number of mitochondrial genomic templates.

### Mitochondrial function is altered in SIN3-deficient cells

**Figure 3:**
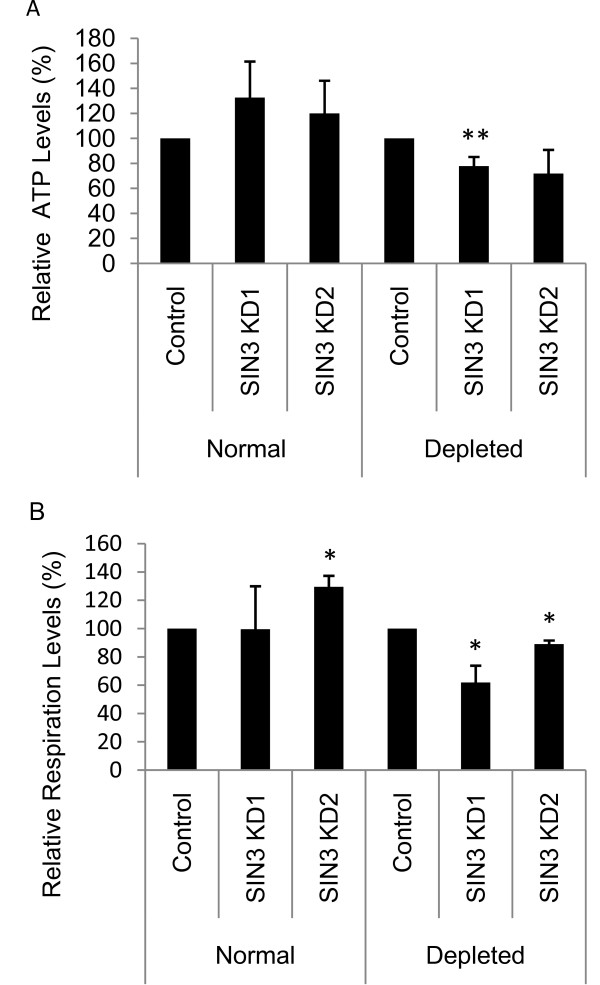
**ATP levels and respiration are affected in S2 cells following loss of SIN3 in nutrient depleted conditions**. (A) ATP levels of S2 cells grown in normal or depleted media with and without SIN3. Error bars represent standard error (n = 3-5). **p = 0.02. ATP present in whole cell extracts prepared from control and SIN3-deficient cells was quantified using the bioluminescent method. (B) Relative respiration levels in S2 cells grown in normal or depleted media with and without SIN3 (n = 3). Error bars represent standard error. *p < 0.05.

Following loss of SIN3, the expression of nuclear and mitochondrial genes encoding proteins important for glycolysis, the citric acid cycle, fatty acid oxidation and oxidative phosphorylation is increased (Figure 1B). Glycolysis, the citric acid cycle and fatty acid oxidation produce NADH and FADH_2 _that feed into oxidative phosphorylation resulting in ATP production. Since transcription of mitochondrial encoded genes can be correlated with ATP levels [30,31] we asked whether ATP levels are increased in SIN3-deficient cells. The relative amount of ATP present in extracts from control and SIN3-deficient S2 cells was determined. We found that while ATP levels were elevated in SIN3-deficient cells relative to the control samples, the increase was not statistically significant (Figure 3A). Proliferating cells grown in culture often rely more heavily upon glycolysis rather than oxidative phosphorylation for ATP production [32]. In some tissue culture cell lines, oxidative phosphorylation has been shown to be inhibited by excess glucose [33]. In an effort to promote oxidative phosphorylation in *Drosophila *S2 cells, we grew the cells in media diluted 1:4 with PBS (depleted) for 24 hours prior to the ATP assay. Under these conditions, SIN3-deficient cells exhibited a decrease in ATP levels relative to the control cells (Figure 3A). These data suggest that when cells are cultured under conditions that favor ATP generation by oxidative phosphorylation rather than by glycolysis, loss of SIN3 results in lowered ATP production compared to that in wild type cells.

We also monitored respiration rate in the SIN3-deficient cells relative to controls. Grown under normal culture conditions, we observed little change in respiration using one dsRNA target sequence and an increase using the other sequence (Figure 3B). In the depleted condition, consistent with the lowered ATP levels, both sets of SIN3-depleted cells had lower respiration rates compared to control cells (Figure 3B). Taken together, the ATP and respiration data suggest that loss of SIN3 leads to changes in gene transcription that affects mitochondrial function.

### Yeast Sin3 mutants exhibit defects in ATP production and respiration

**Figure 4:**
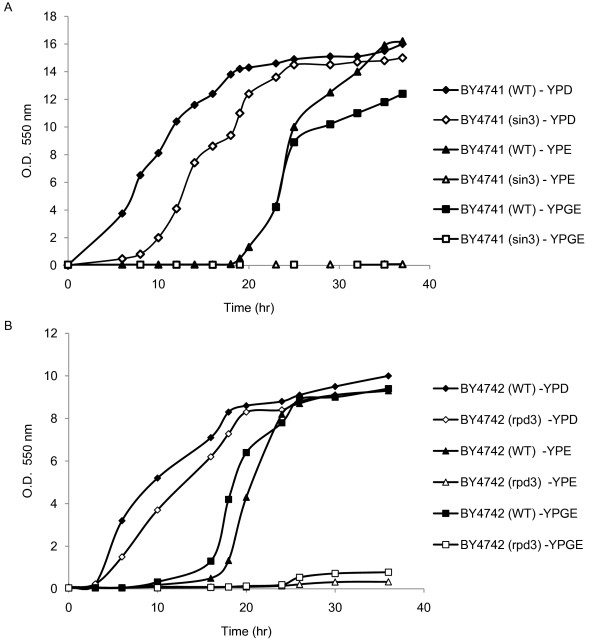
***S. cerevisiae SIN3 *and *RPD3 *are critical for growth in media prepared with non-fermentable carbon sources**. Wild type (BY4741 WT), *sin3 *null mutant (BY4741 sin3) (A), wild type (BY4742 WT) and *rpd3 *null mutant (BY4742 rpd3) (B) cells were grown in 5 ml of YPD, washed twice with water, inoculated into media containing glucose (YPD), ethanol (YPE) and glycerol and ethanol (YPGE) as sole carbon sources and incubated at 30°C. Absorbance at 550 nm was measured at the indicated times. Representative results of single growth curve assays are shown. A minimum of three independent trials was performed and all produced similar results.

Unlike in *Drosophila, Saccharomyces cerevisiae SIN3 *is not an essential gene [34]. Gene expression profiles of null mutants revealed that yeast Sin3 (ySin3) regulates genes involved in carbon metabolism [35,36]. We hypothesized that as ySin3 regulates expression of genes encoding proteins that function in the mitochondria, the *sin3 *mutant may display altered ability to grow in media prepared with non-fermentable carbon sources. To test our hypothesis, we monitored the growth rates of wild type and *sin3 *mutant cells in glucose-containing fermentable media (YPD) and in non-fermentable media. Glucose is the preferred carbon source for yeast, which produce ATP through glycolysis and fermentation. In the absence of glucose and in the presence of other carbon sources, including glycerol and ethanol, ATP production can only occur through the citric acid cycle and oxidative phosphorylation requiring functional mitochondria [37]. The *sin3 *mutant displayed a slightly delayed growth phenotype when cultured in YPD (Figure 4A and Additional file 2). This growth defect is possibly due to a cell cycle delay in the G2 phase of the cell cycle. *SIN3 *was recently found to be important for cell cycle progression as the percentage of cells in G2 phase was elevated in the null mutant relative to the control strain [38]. In striking contrast to the data obtained on glucose-containing media, the *sin3 *mutant grew very poorly in each of the non-fermentable media tested (Figure 4A, Additional file 2).

As *SIN3 *was not previously identified in global screens for factors required for respiratory activity [39-42], we wished to verify our findings using a *sin3 *mutant having a different genetic background. Two additional independent *sin3 *mutants grew very poorly on non-fermentable carbon sources as compared to their wild type counterpart strains (Additional file 3). Very poor growth of the mutants was also noted when the strains were spread onto solid agar plates containing the respective carbon sources (Additional files 4, 5) Given the poor growth on non-fermentable carbon sources, it is important to verify that the *sin3 *mutant strains are not petite. To verify that the strains contain mitochondrial DNA, the cells were stained with DAPI. Mitochondrial genomes were observed in all mutants indicating the defective growth on non-fermentable carbon is not due to the strains becoming rho^o ^(Additional file 6).

We also tested whether a deletion in yRpd3, the catalytic component of the SIN3 histone deacetyase complex, would affect growth in non-fermentable carbon. Like *SIN3*, *RPD3 *is a non-essential gene [43]. As expected, we determined that yRpd3 was dispensable for growth in glucose-containing YPD (Figure 4B, Additional file 7). Similar to the *sin3 *mutants, the *rpd3 *mutant grew very poorly in liquid media and on solid agar plates containing non-fermentable carbon sources (Figure 4B, Additional file 7). Staining of mitochondrial DNA in the *rpd3 *mutant verified that the strain was not rho^o ^(Additional file 6). Taken together, these results clearly demonstrate that yeast *SIN3 *and *RPD3 *are critical for mitochondrial function.

**Figure 5:**
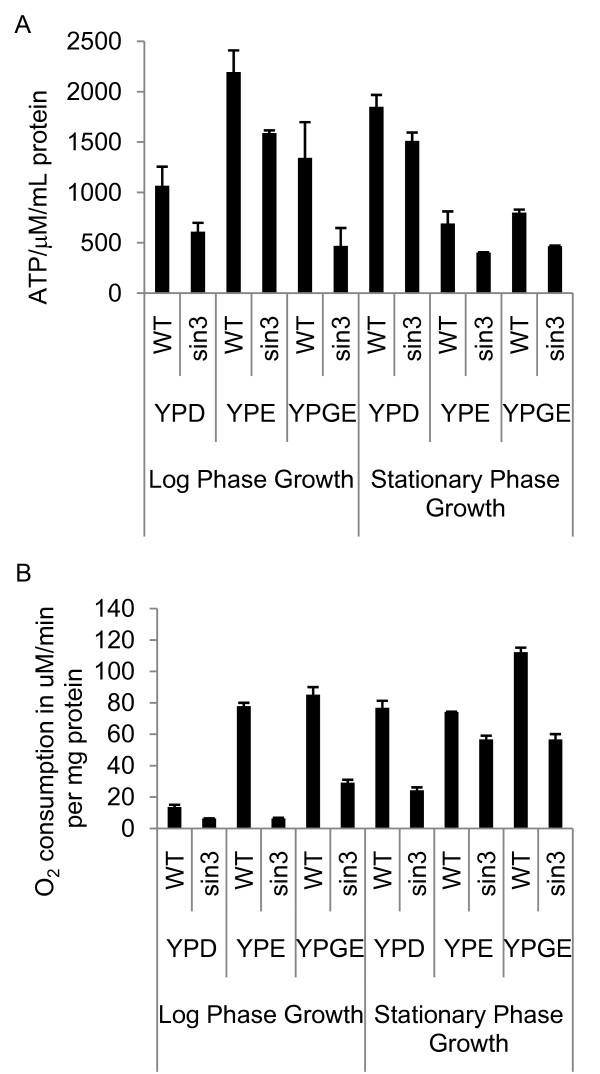
**ATP levels and respiration are decreased in yeast *sin3 *null mutants**. (A) ATP levels of yeast grown in YPD and non-fermentable carbon sources measured as ATP/μM/mg protein. Error bars represent standard error (n = 3). ATP present in whole cell extracts prepared from control and SIN3-deficient yeast was quantified using the bioluminescent method. (B) Respiration levels of yeast grown in YPD and non-fermentable carbon sources measured as O_2 _consumption in μM/min per mg of protein. Error bars represent standard deviation.

To further analyze the yeast *sin3 *mutant for mitochondrial activity, we monitored ATP levels and respiration rates in wild type and *sin3 *strains. We performed these assays using cells grown in glucose and non-fermentable media. Samples were collected from cells in both log and stationary phases of growth. During log phase, yeast produce ATP primarily through fermentation. As glucose in the media becomes exhausted, cells become adapted to mitochondrial respiration, a metabolic process known as the diauxic shift [44]. The respiration rate of wild type cells isolated in stationary phase was higher relative to the rate in cells that were still in the log phase of growth, indicating the switch to aerobic respiration seen in the diauxic shift (Figure 5B). In all media and in both phases of growth, the *sin3 *null mutant showed lower levels of ATP (Figure 5A). Consistent with the lowered levels of ATP, the respiration rates were also decreased in the *sin3 *mutants relative to wild type cells (Figure 5B). The findings that yeast *sin3 *mutants are unable to grow on non-fermentable carbon sources and show lowered ATP and reduced oxygen consumption indicate that ySin3 is important for mitochondrial function. The observations that both yeast and *Drosophila *SIN3 affect mitochondrial activity imply an evolutionarily conserved role for SIN3 in the control of cellular energy production.

## Discussion

We previously established that the expression of numerous nuclear encoded mitochondrial genes was up-regulated in *Drosophila *tissue culture cells following the loss of the corepressor SIN3 [5]. In research described in this current work, we first determined that the changes in gene expression following loss of SIN3 are unlikely due to off target effects of SIN3 RNAi. Targeting two different regions of the SIN3 mRNA results in similar changes in gene expression, indicating that the changes are likely due to loss of SIN3 and not to an effect of another gene that contains a short similar sequence. In addition to the changes in expression of the nuclear encoded genes, loss of SIN3 results in an up-regulation of mitochondrial encoded genes. Although we observe changes in gene expression, we find that mitochondrial genome copy number is unaltered. The changes in gene expression prompted us to examine mitochondrial function in SIN3-deficient cells. SIN3-deficient cells show changes in ATP levels and respiration rates, suggesting that the increases in gene expression result in altered oxidative phosphorylation capacity. A necessary role of SIN3 for normal mitochondrial function is supported by the finding that yeast *sin3 *mutants are unable to grow on non-fermentable carbon sources, conditions which require active mitochondria for ATP production.

Consistent with numerous published reports, we have determined that loss of ySin3 is dispensable for fermentative growth [9]. In contrast, ySin3 is critical for growth when respiration is required (Figure 4A, Additional files 2, 3, 4 and 5). Genome wide studies have been conducted to identify yeast mutant strains that have the ability to grow on YPD and exhibit reduced growth on non-fermentable carbon sources but failed to identify *SIN3 *[39-42]. Several possible explanations may account for false negatives in global screens, three of which are discussed by the authors of one of the global screen studies [40]. First, methods of analysis for growth and scoring differ. Second, there may be phenotypic plasticity between mutants in different strain backgrounds. Third, the yeast deletion collections are known to accumulate errors, and it is well-established that deletion strains must first be tested to ensure that the deletion is correct. Obviously, this cannot be done when carrying out global screens. In addition, in the global study of Steinmetz *et al. *[39], the *sin3 *mutant did not grow well when cultured in any of the different carbon sources under the assay conditions tested, including YPD. Pools of mutants were cultured in various media and growth of individual mutant strains of the pool was compared to average growth in the culture. It is possible that the *sin3 *strain was not able to grow in any of the assay conditions in such pools of mutants. All of the above are potential reasons as to why *SIN3 *was not identified in a global screen for genes required for respiratory activity. In this study we demonstrate that, when assayed as an individual strain, the *sin3 *mutant grows very poorly in non-fermentable media, indicating that ySin3 is required when cells are forced to respire. Consistent with this data, *sin3 *mutants have lower levels of ATP and decreased respiration rates.

Work from multiple laboratories has indicated that there are many similarities between yeast and metazoan SIN3, including the fact that SIN3 acts as a global regulator of transcription and that it functions in the context of the SIN3 histone deactylase complex [9]. One major reported difference between yeast and metazoan SIN3 is the requirement of this gene for viability. ySin3 is dispensable for growth while metazoan SIN3 is an essential gene [9,45]. Analysis of conditional knockdown of *Drosophila *SIN3 mutants suggests that SIN3-deficiency leads to death due to defects in cell cycle progression rather than due to alteration of a specific developmental pathway [8]. In this work, we have found that ySin3 is required for cell growth when mitochondrial function is necessary for ATP production. It is thus possible that, rather than being essential for some specific process of development, the metazoan requirement for SIN3 is due to its requirement for normal mitochondrial activity. We hypothesize that loss of SIN3 leads to a defect in mitochondrial function that in turn leads to a defect in cell cycle progression. Links between cell cycle control and mitochondrial activity are well established. Myc: Max heterodimers positively regulate the cell cycle while Rb-E2F complexes repress genes necessary for cell cycle progression [46,47]. Both sets of factors have been linked to regulation by NRF-1, a key transcription activator of nuclear encoded mitochondrial genes [48,49]. SIN3 has been linked to both the Max transcription network and to regulation by Rb, supporting the hypothesis that SIN3 regulation of mitochondrial biogenesis and activity is linked to cell cycle control [18,50]. It was recently shown in mammalian cells that changes in mitochondrial morphology are crucial for cell cycle control. At the G_1_-S checkpoint mitochondria are organized as a large fused network with higher mitochondrial membrane potentials, increased ATP levels and increased respiration rates, whereas in other stages of the cell cycle, mitochondria are predominantly in the fission state [51]. Lower ATP levels and respiration rates caused by SIN3 knockdown (Figure 3, depleted condition) may thus interfere with cell cycle progression at the G_1_-S transition.

In addition to its role in gene regulation, SIN3 might directly regulate energy metabolism as a participant in cell signaling. There is clear evidence that signaling pathways regulate mitochondrial respiration and energy levels, e.g., the cAMP and inflammatory pathways lead to phosphorylation of cytochrome *c *oxidase, the terminal enzyme of the electron transport chain, followed by changes in respiration and ATP levels [52,53]. SIN3 might exert a stimulatory signal required for normal mitochondrial function. The observation that reduction in SIN3 results in decreased ATP levels and respiration is perhaps surprising in light of the finding that expression of many genes encoding proteins with mitochondrial function are up-regulated. Up-regulation of nuclear encoded mitochondrial genes upon SIN3 knockdown may therefore be a compensatory response to an increase in the mitochondrial component due to dysfunctional energy metabolism, as is seen when yeast must adapt to mitochondrial respiration in full dependence on glycolysis (Figure 4, YPD). Alternatively, mis-coordination of mitochondrial gene expression in the SIN3-deficient cells may result in the observed altered mitochondrial function because loss of SIN3 leads to an increase in expression of many but not all genes involved in mitochondrial processes [5]. As has been previously suggested, an imbalance in the level of the proteins present in complexes important for energy production may result in mitochondrial malfunction [48,54]. Finally, key links between regulation of acetylation and metabolism are highlighted in recently published data indicating that ATP-citrate lyase is important for maintenance of histone acetylation levels in growing cells [55]. It is possible that SIN3 knockdown affects acetylation resulting not only in changes in gene expression but also altered pools of key metabolites and an inappropriate response of the mitochondria to these abnormal signals. It will be of interest to further investigate the relationship between histone acetylation and mitochondrial function to better understand control of cellular metabolism.

## Conclusion

In summary, mitochondrial activity was affected following loss of SIN3 in both *Drosophila *cultured cells and in yeast. This abnormal mitochondrial function may lead to cell cycle defects and thus affect cell and organism viability.

## Methods

### Cell culture and RNA interference

*Drosophila *S2 cell culture and RNAi was carried out as previously described [56]. RNAi was performed using dsRNA corresponding to two distinct regions of SIN3 mRNA. SIN3 RNAi knockdown #1 dsRNA is targeted against a sequence encoded in exon 3. The construction of targeting sequence #1 in pCRII-Topo vector (Invitrogen) is described in [56]. SIN3 RNAi knockdown #2 dsRNA is targeted against exon 4. The sequences in the pCRII-Topo vector were generated using the following primer set (oriented 5' to 3') TCACCAAGTTATGGCATTCC and CTTAGGTACTGTGGACTGCG. Mock RNAi treated cells served as the control.

### RT-PCR analysis

Total RNA was extracted from tissue culture cells using the RNeasy purification kit (Qiagen). cDNA was generated from total RNA using the ImProm-II Reverse Transcription System (Promega) with random hexamers. The cDNA was used as template in a quantitative real-time PCR (qPCR) assay. The analysis was performed using ABsolute SYBR Green ROX master mix (Fisher Scientific) and carried out in a Stratagene Mx3005P real-time thermocycler. Primers used are listed in Additional file 1. Primers were designed using PRIMER3 primer design software [57], except primers for TFAM, TFB1, TFB2, TTF, CG4464 which were described previously by Dubessay, *et al. *[58]. TAF1 was used to normalize cDNA amounts in the comparative analysis.

### Western blot analysis

Western blot analysis was performed in accordance with standard protocols [59]. To prepare whole cell extracts, cells were pelleted by centifugation and then lysed in Laemmli sample buffer (Bio-Rad) at a concentration of 1.5 × 10^4 ^cell/μl of buffer. Protein concentration was determined using the DC protein assay reagent (Bio-Rad) according to the manufacturer's protocol. Protein extract (15 to 20 μg) was fractionated by sodium dodecyl sulfate (SDS) 8% polyacrylamide gel electrophoresis (PAGE), transferred to a polyvinylidene difluoride membrane, (PVDF; Pall), and probed with immunoglobulin G (IgG) purified polyclonal rabbit antibodies against SIN3 (1:2000) [60], followed by donkey anti-rabbit horseradish peroxidase-conjugated IgG (1:3000) (GE Healthcare), and detected with enhanced chemiluminescence reagents (GE Healthcare). The blots were subsequently probed with monoclonal mouse antibody against β-tubulin (1:1000) (Sigma), followed by sheep anti-mouse horseradish peroxidase-conjugated IgG (1:3000) (GE Healthcare) as a loading control.

### Genome copy number determination

Genomic DNA was prepared from tissue culture cells according to standard protocol [61]. The isolated DNA was used as a template in a qPCR assay. The analysis was performed using ABsolute SYBR Green ROX master mix (Fisher Scientific) and carried out in a Stratagene Mx3005P real-time thermocycler. Primers used amplified three nuclear and four mitochondrial genomic regions and are listed in Additional file 1. CG11784 (glutathione transferase) genomic sequence was used to normalize the DNA amounts in the reaction.

### Determination of cellular ATP levels

*Drosophila *S2 or yeast cells were grown in culture to yield approximately 1 × 10^7 ^cells. Cells were transferred to 15 ml Falcon tubes, pelleted by quick spin at 805 × *g*, rinsed with 1.5 ml of PBS, and re-pelleted in 2 ml microcentrifuge tubes at 16,100 × *g *for 15 sec. After discarding PBS, cells were quick frozen in liquid nitrogen and stored at -80°C until measurement. Cell pellets were supplemented with 300 μl of boiling buffer (100 mM Tris, pH 7.75, 4 mM EDTA) and immediately transferred to a boiling water bath and lysed for 2 min, put on ice, and solubilized by ultrasonication. ATP concentration was determined using the ATP bioluminescence assay kit HS II (Roche Applied Science) according to the manufacturer's protocol. Data were standardized to the protein concentration, as was determined with the DC protein assay kit (Bio-Rad). For protein determination of the yeast samples, 100 μl of a 20% SDS solution was added and ultrasonicated again to completely solubilize proteins.

### Respiration assay

Cells were collected and protein concentration was determined with the DC protein assay kit (Bio-Rad). Cells were then washed with PBS, and resuspended in Schneider's *Drosophila *media for S2 cells (GIBCO, Invitrogen) or complex media for liquid yeast cultures containing yeast extract (1%), peptone (2%), and either glucose (2%), ethanol (1%) or ethanol (1%) plus glycerol (2%) (Difco, Fisher Scientific) to 1 mg/ml final protein concentration. Oxygen consumption of the cells was measured in a closed 200 μl chamber equipped with a micro Clark-type oxygen electrode (Oxygraph system, Hansatech) at 25°C and analyzed with Oxygraph software. 500 μM KCN was added at the end of the each measurement to inhibit cytochrome c oxidase. Non-cytochrome *c *oxidase-based respiration was subtracted to determine oxygen consumption rates. Respiration was defined as oxygen consumed (μM/min per mg protein).

### Yeast growth assay

Glucose, yeast extract and peptone were obtained from Difco. Glycerol was obtained from Fisher Scientific. All chemicals used were reagent grade or better. Yeast cultures were maintained in 15% glycerol at -80°C for long term storage and on YPD plates for short term storage. Complex media for liquid cultures contained yeast extract (1%), peptone (2%), and either glucose (2%), ethanol (1%) or ethanol (1%) plus glycerol (2%). For the liquid growth curve assay, 5 ml of YPD liquid media was inoculated with a single colony from a solid agar YPD plate and the yeast strains were grown to stationary phase by overnight culture. The volume of stationary culture needed to make 25 ml culture starting at 0.05-0.06 OD (at 550 nm) was transferred to a 1.5 ml tube and the cells pelleted by centrifugation. The yeast pellet was washed 2 times with 1 ml of ddH2O and then resuspended in 25 ml of media containing the different carbon sources. The cultures were incubated at 30°C with shaking at 230-270 rpm. The absorbance at 550 nm was determined at the indicated times to follow the growth of the culture. For the plate assay, strains were grown to stationary phase as above, counted on a hemocytometer, washed 2 times with 1 ml of ddH2O and 300 cells were spread onto respective agar plates. The plates were incubated at 30°C for 3-6 days. The deletion mutants were verified by PCR using the following primers (oriented 5' to 3'): Sin3, ATCACCCTCGCCCTAAATC and CCGGTGTGTCTATTGCCTG; Rpd3, TTCGGCATCCCTAGGTGGTGATT and TCTGCTGTCGTGTTACAGTGTGGT.

Strains used:

BY4741 (WT) *MAT a, his 3Δ1, leu 2Δ0, met 15Δ0, ura3Δ0*

BY4741 *sin3Δ MAT a, his *3Δ1, *leu 2Δ0, met 15Δ0, ura3Δ0, sin3Δ::KanMX4*

FY23 (WT) *MAT a, ura3-52, trp1Δ63, leu2Δ1*

FY23 *sin3*Δ *MAT a, ura3-52, trp1Δ63, leu2Δ1, sin3Δ::KanMX4*

BY4733 (WT) *MAT a, his3Δ200, trp1Δ63, leu2Δ0, met15Δ0, ura3Δ0*

BY4733 *sin3*Δ *MAT a, his3Δ200, trp1Δ63, leu2Δ0, met15Δ0, ura3Δ0, sin3Δ::KanMX4*

BY4742 (WT) *MATΔ, his3Δ1, leu2Δ0, lys2Δ0, ura3Δ0*

BY4742 *rpd3Δ MATΔ, his3Δ1, leu2Δ0, lys2Δ0, ura3Δ0, rpd3Δ::KanMX4*

### Yeast DAPI staining

To stain nuclear and mitochondrial DNA, yeast cells were cultured to the mid-log phase, fixed in 70% ethanol at room temperature for 30 min, washed once with ddH2O, and stained with 500 ng/ml DAPI (Sigma) for 5 min. All microscopy was performed using a Zeiss Axioscope 2 fluorescence microscope. Images were acquired using Olympus QCapture2 software (1000× magnification).

## Authors' contributions

VLB carried out the RT-PCR, Western blot, growth curve and DAPI staining assays and prepared samples for respiration and ATP analysis. BSS performed RT-PCR and genome copy number assays. IL analyzed respiration levels. MH conceived of the respiration and ATP assays, analyzed ATP levels, and helped to draft the manuscript. LAP conceived of the study and participated in its design and coordination and drafted the manuscript. All authors read and approved the final manuscript.

## Supplementary Material

Additional file 1 **Primer pairs used in this study**. This table lists all primer pairs used for qPCR gene expression analysis. Click here for file

Additional file 2 ***sin3 *mutant cells grow very poorly on solid agar plates containing non-fermentable carbon sources**. This file contains images showing the growth of wild type and a *sin3 *mutant on solid agar plates containing YPD or non-fermentable carbon sources. Click here for file

Additional file 3 **Additional *S. cerevisiae *strains demonstrate ySin3 is critical for growth in media prepared with non-fermentable carbon sources**. This file shows growth curves for two additional *S. cerevisiae *strains, with differing genetic backgrounds, in media prepared with YPD or non-fermentable carbon sources. Click here for file

Additional file 4 **A second *S. cerevisiae *strain demonstrates ySin3 is critical for growth on media prepared with non-fermentable carbon sources**. This file contains images showing the growth of wild type and a *sin3 *mutant of different genetic background on solid agar plates containing YPD or non-fermentable carbon sources. Click here for file

Additional file 5 **A third *S. cerevisiae *strain demonstrates ySin3 is critical for growth on media prepared with non-fermentable carbon sources**. This file contains images showing the growth of wild type and a *sin3 *mutant of different genetic background on solid agar plates containing YPD or non-fermentable carbon sources. Click here for file

Additional file 6 **Mitochondrial genomes are observed in all *sin3 *and *rpd3 *mutants**. This file contains images of DAPI staining of nuclear and mitochondrial DNA in wild type and *sin3 *and *rpd3 *mutant cells. Click here for file

Additional file 7 ***rpd3 *mutant cells grow very poorly on solid agar plates containing non-fermentable carbon sources**. This file contains images showing the growth of wild type and an *rpd3 *mutant on solid agar plates containing YPD or non-fermentable carbon sources. Click here for file

## References

[B1] Scarpulla RC Nuclear control of respiratory chain expression by nuclear respiratory factors and PGC-1-related coactivator Ann N Y Acad Sci 2008 1147 321 334 19076454 10.1196/annals.1427.006 PMC2853241

[B2] Scarpulla RC Nuclear activators and coactivators in mammalian mitochondrial biogenesis Biochim Biophys Acta 2002 1576 1-2 1 14 12031478 10.1016/s0167-4781(02)00343-3

[B3] Hughes TR Marton MJ Jones AR Roberts CJ Stoughton R Armour CD Bennett HA Coffey E Dai H He YD Functional discovery via a compendium of expression profiles Cell 2000 102 1 109 126 10.1016/S0092-8674(00)00015-5 10929718

[B4] Mootha VK Bunkenborg J Olsen JV Hjerrild M Wisniewski JR Stahl E Bolouri MS Ray HN Sihag S Kamal M Integrated analysis of protein composition, tissue diversity, and gene regulation in mouse mitochondria Cell 2003 115 5 629 640 10.1016/S0092-8674(03)00926-7 14651853

[B5] Pile LA Spellman PT Katzenberger RJ Wassarman DA The SIN3 deacetylase complex represses genes encoding mitochondrial proteins: implications for the regulation of energy metabolism J Biol Chem 2003 278 39 37840 37848 10.1074/jbc.M305996200 12865422

[B6] Pennetta G Pauli D The Drosophila Sin3 gene encodes a widely distributed transcription factor essential for embryonic viability Development genes and evolution 1998 208 9 531 536 10.1007/s004270050212 9799435

[B7] Neufeld TP Tang AH Rubin GM A genetic screen to identify components of the sina signaling pathway in Drosophila eye development Genetics 1998 148 1 277 286 9475739 10.1093/genetics/148.1.277 PMC1459784

[B8] Sharma V Swaminathan A Bao R Pile LA Drosophila SIN3 is required at multiple stages of development Dev Dyn 2008 237 10 3040 3050 10.1002/dvdy.21706 18816856

[B9] Silverstein RA Ekwall K Sin3: a flexible regulator of global gene expression and genome stability Current genetics 2005 47 1 1 17 10.1007/s00294-004-0541-5 15565322

[B10] Zhang Y Iratni R Erdjument-Bromage H Tempst P Reinberg D Histone deacetylases and SAP18, a novel polypeptide, are components of a human Sin3 complex Cell 1997 89 3 357 364 10.1016/S0092-8674(00)80216-0 9150135

[B11] Hassig CA Fleischer TC Billin AN Schreiber SL Ayer DE Histone deacetylase activity is required for full transcriptional repression by mSin3A Cell 1997 89 3 341 347 10.1016/S0092-8674(00)80214-7 9150133

[B12] Laherty CD Yang WM Sun JM Davie JR Seto E Eisenman RN Histone deacetylases associated with the mSin3 corepressor mediate mad transcriptional repression Cell 1997 89 3 349 356 10.1016/S0092-8674(00)80215-9 9150134

[B13] Chen J Shi X Padmanabhan R Wang Q Wu Z Stevenson SC Hild M Garza D Li H Identification of novel modulators of mitochondrial function by a genome-wide RNAi screen in Drosophila melanogaster Genome Res 2008 18 1 123 136 10.1101/gr.6940108 18042644 PMC2134776

[B14] Cho Y Griswold A Campbell C Min KT Individual histone deacetylases in Drosophila modulate transcription of distinct genes Genomics 2005 86 5 606 617 10.1016/j.ygeno.2005.07.007 16137856

[B15] Foglietti C Filocamo G Cundari E De Rinaldis E Lahm A Cortese R Steinkuhler C Dissecting the biological functions of Drosophila histone deacetylases by RNA interference and transcriptional profiling J Biol Chem 2006 281 26 17968 17976 10.1074/jbc.M511945200 16632473

[B16] Mottus R Sobel RE Grigliatti TA Mutational analysis of a histone deacetylase in Drosophila melanogaster: missense mutations suppress gene silencing associated with position effect variegation Genetics 2000 154 2 657 668 10655219 10.1093/genetics/154.2.657 PMC1460943

[B17] Orian A van Steensel B Delrow J Bussemaker HJ Li L Sawado T Williams E Loo LW Cowley SM Yost C Genomic binding by the Drosophila Myc, Max, Mad/Mnt transcription factor network Genes Dev 2003 17 9 1101 1114 10.1101/gad.1066903 12695332 PMC196053

[B18] Ayer DE Lawrence QA Eisenman RN Mad-Max transcriptional repression is mediated by ternary complex formation with mammalian homologs of yeast repressor Sin3 Cell 1995 80 5 767 776 10.1016/0092-8674(95)90355-0 7889570

[B19] Semenza GL Regulation of mammalian O2 homeostasis by hypoxia-inducible factor 1 Annu Rev Cell Dev Biol 1999 15 551 578 10.1146/annurev.cellbio.15.1.551 10611972

[B20] Mahon PC Hirota K Semenza GL FIH-1: a novel protein that interacts with HIF-1alpha and VHL to mediate repression of HIF-1 transcriptional activity Genes Dev 2001 15 20 2675 2686 10.1101/gad.924501 11641274 PMC312814

[B21] Evans MJ Scarpulla RC Interaction of nuclear factors with multiple sites in the somatic cytochrome c promoter. Characterization of upstream NRF-1, ATF, and intron Sp1 recognition sequences J Biol Chem 1989 264 24 14361 14368 2547796

[B22] Zhang Y Dufau ML Silencing of transcription of the human luteinizing hormone receptor gene by histone deacetylase-mSin3A complex J Biol Chem 2002 277 36 33431 33438 10.1074/jbc.M204417200 12091390

[B23] Ufer C Borchert A Kuhn H Functional characterization of cis- and trans-regulatory elements involved in expression of phospholipid hydroperoxide glutathione peroxidase Nucleic Acids Res 2003 31 15 4293 4303 10.1093/nar/gkg650 12888488 PMC169948

[B24] Puigserver P Spiegelman BM Peroxisome proliferator-activated receptor-gamma coactivator 1 alpha (PGC-1 alpha: transcriptional coactivator and metabolic regulator Endocr Rev 2003 24 1 78 90 10.1210/er.2002-0012 12588810

[B25] Mandard S Muller M Kersten S Peroxisome proliferator-activated receptor alpha target genes Cell Mol Life Sci 2004 61 4 393 416 10.1007/s00018-003-3216-3 14999402 PMC11138883

[B26] Jones DC Ding X Daynes RA Nuclear receptor peroxisome proliferator-activated receptor alpha (PPARalpha) is expressed in resting murine lymphocytes. The PPARalpha in T and B lymphocytes is both transactivation and transrepression competent J Biol Chem 2002 277 9 6838 6845 10.1074/jbc.M106908200 11726654

[B27] Deroo BJ Hewitt SC Peddada SD Korach KS Estradiol regulates the thioredoxin antioxidant system in the mouse uterus Endocrinology 2004 145 12 5485 5492 10.1210/en.2004-0471 15345672

[B28] Ma Y Creanga A Lum L Beachy PA Prevalence of off-target effects in Drosophila RNA interference screens Nature 2006 443 7109 359 363 10.1038/nature05179 16964239

[B29] Garesse R Vallejo CG Animal mitochondrial biogenesis and function: a regulatory cross-talk between two genomes Gene 2001 263 1-2 1 16 10.1016/S0378-1119(00)00582-5 11223238

[B30] DasGupta SF Rapoport SI Gerschenson M Murphy E Fiskum G Russell SJ Chandrasekaran K ATP synthesis is coupled to rat liver mitochondrial RNA synthesis Mol Cell Biochem 2001 221 1-2 3 10 10.1023/A:1010812128765 11506183

[B31] Enriquez JA Fernandez-Silva P Perez-Martos A Lopez-Perez MJ Montoya J The synthesis of mRNA in isolated mitochondria can be maintained for several hours and is inhibited by high levels of ATP Eur J Biochem 1996 237 3 601 610 10.1111/j.1432-1033.1996.0601p.x 8647103

[B32] Golshani-Hebroni SG Bessman SP Hexokinase binding to mitochondria: a basis for proliferative energy metabolism J Bioenerg Biomembr 1997 29 4 331 338 10.1023/A:1022442629543 9387093

[B33] Ibsen KH The Crabtree effect: a review Cancer Res 1961 21 829 841 13717358

[B34] Wang H Clark I Nicholson PR Herskowitz I Stillman DJ The Saccharomyces cerevisiae SIN3 gene, a negative regulator of HO, contains four paired amphipathic helix motifs Mol Cell Biol 1990 10 11 5927 5936 2233725 10.1128/mcb.10.11.5927-5936.1990 PMC361389

[B35] Bernstein BE Tong JK Schreiber SL Genomewide studies of histone deacetylase function in yeast Proc Natl Acad Sci USA 2000 97 25 13708 13713 10.1073/pnas.250477697 11095743 PMC17640

[B36] Fazzio TG Kooperberg C Goldmark JP Neal C Basom R Delrow J Tsukiyama T Widespread collaboration of Isw2 and Sin3-Rpd3 chromatin remodeling complexes in transcriptional repression Mol Cell Biol 2001 21 19 6450 6460 10.1128/MCB.21.19.6450-6460.2001 11533234 PMC99792

[B37] Johnson M Carlson M Jones EW, Pringle JR, Broach JR Regulation of Carbon and Phosphate Utilization The Molecular and Cellular Biology of the Yeast Saccharojyces: Gene expression 1992 2

[B38] White MA Riles L Cohen BA A systematic screen for transcriptional regulators of the yeast cell cycle Genetics 2009 181 2 435 446 10.1534/genetics.108.098145 19033152 PMC2644938

[B39] Steinmetz LM Scharfe C Deutschbauer AM Mokranjac D Herman ZS Jones T Chu AM Giaever G Prokisch H Oefner PJ Systematic screen for human disease genes in yeast Nat Genet 2002 31 4 400 404 12134146 10.1038/ng929

[B40] Merz S Westermann B Genome-wide deletion mutant analysis reveals genes required for respiratory growth, mitochondrial genome maintenance and mitochondrial protein synthesis in Saccharomyces cerevisiae Genome Biol 2009 10 9 R95 10.1186/gb-2009-10-9-r95 19751518 PMC2768984

[B41] Dimmer KS Fritz S Fuchs F Messerschmitt M Weinbach N Neupert W Westermann B Genetic basis of mitochondrial function and morphology in Saccharomyces cerevisiae Mol Biol Cell 2002 13 3 847 853 10.1091/mbc.01-12-0588 11907266 PMC99603

[B42] Luban C Beutel M Stahl U Schmidt U Systematic screening of nuclear encoded proteins involved in the splicing metabolism of group II introns in yeast mitochondria Gene 2005 354 72 79 10.1016/j.gene.2005.03.023 15908144

[B43] Vidal M Gaber RF RPD3 encodes a second factor required to achieve maximum positive and negative transcriptional states in Saccharomyces cerevisiae Mol Cell Biol 1991 11 12 6317 6327 1944291 10.1128/mcb.11.12.6317-6327.1991 PMC361826

[B44] Werner-Washburne M Braun E Johnston GC Singer RA Stationary phase in the yeast Saccharomyces cerevisiae Microbiol Rev 1993 57 2 383 401 8393130 10.1128/mr.57.2.383-401.1993 PMC372915

[B45] Grandinetti KB David G Sin3B: an essential regulator of chromatin modifications at E2F target promoters during cell cycle withdrawal Cell cycle (Georgetown, Tex) 2008 7 11 1550 1554 18469515 10.4161/cc.7.11.6052

[B46] Blais A Dynlacht BD E2F-associated chromatin modifiers and cell cycle control Current opinion in cell biology 2007 19 6 658 662 10.1016/j.ceb.2007.10.003 18023996 PMC3586187

[B47] Eilers M Eisenman RN Myc's broad reach Genes Dev 2008 22 20 2755 2766 10.1101/gad.1712408 18923074 PMC2751281

[B48] Morrish F Hockenbery D Myc's mastery of mitochondrial mischief Cell cycle (Georgetown, Tex) 2003 2 1 11 13 12695675 10.4161/cc.2.1.275

[B49] Cam H Balciunaite E Blais A Spektor A Scarpulla RC Young R Kluger Y Dynlacht BD A common set of gene regulatory networks links metabolism and growth inhibition Mol Cell 2004 16 3 399 411 10.1016/j.molcel.2004.09.037 15525513

[B50] Rayman JB Takahashi Y Indjeian VB Dannenberg JH Catchpole S Watson RJ te Riele H Dynlacht BD E2F mediates cell cycle-dependent transcriptional repression in vivo by recruitment of an HDAC1/mSin3B corepressor complex Genes Dev 2002 16 8 933 947 10.1101/gad.969202 11959842 PMC152357

[B51] Mitra K Wunder C Roysam B Lin G Lippincott-Schwartz J A hyperfused mitochondrial state achieved at G1-S regulates cyclin E buildup and entry into S phase Proc Natl Acad Sci USA 2009 106 29 11960 11965 10.1073/pnas.0904875106 19617534 PMC2710990

[B52] Lee I Salomon AR Ficarro S Mathes I Lottspeich F Grossman LI Huttemann M cAMP-dependent tyrosine phosphorylation of subunit I inhibits cytochrome c oxidase activity J Biol Chem 2005 280 7 6094 6100 10.1074/jbc.M411335200 15557277

[B53] Samavati L Lee I Mathes I Lottspeich F Huttemann M Tumor necrosis factor alpha inhibits oxidative phosphorylation through tyrosine phosphorylation at subunit I of cytochrome c oxidase J Biol Chem 2008 283 30 21134 21144 10.1074/jbc.M801954200 18534980 PMC3258931

[B54] Morrish F Giedt C Hockenbery D c-MYC apoptotic function is mediated by NRF-1 target genes Genes Dev 2003 17 2 240 255 10.1101/gad.1032503 12533512 PMC195978

[B55] Wellen KE Hatzivassiliou G Sachdeva UM Bui TV Cross JR Thompson CB ATP-citrate lyase links cellular metabolism to histone acetylation Science 2009 324 5930 1076 1080 10.1126/science.1164097 19461003 PMC2746744

[B56] Pile LA Schlag EM Wassarman DA The SIN3/RPD3 deacetylase complex is essential for G(2) phase cell cycle progression and regulation of SMRTER corepressor levels Mol Cell Biol 2002 22 14 4965 4976 10.1128/MCB.22.14.4965-4976.2002 12077326 PMC139766

[B57] SDSC Biology Workbench http://seqtool.sdsc.edu

[B58] Dubessay P Garreau-Balandier I Jarrousse AS Fleuriet A Sion B Debise R Alziari S Aging impact on biochemical activities and gene expression of Drosophila melanogaster mitochondria Biochimie 2007 89 8 988 1001 10.1016/j.biochi.2007.03.018 17524546

[B59] Sambrook J Russell DW (eds) Molecular Cloning- A Laboratory Manual 2001 Third New York: CSHL press

[B60] Pile LA Wassarman DA Chromosomal localization links the SIN3-RPD3 complex to the regulation of chromatin condensation, histone acetylation and gene expression Embo J 2000 19 22 6131 6140 10.1093/emboj/19.22.6131 11080159 PMC305822

[B61] Huang AM Rehm EJ Rubin GM Sullivan W, Ashburner M, Hawley RS Recovery of DNA Sequences Flanking P-element Insertions: Inverse PCR and Plasmid Rescue Drosophila Protocols 2000 Cold Spring Harbor, NY: Cold Spring Harbor Laboratory Press 431 432 10.1101/pdb.prot5199 20147142

