# Supplementary material for: Loss of the SIN3 transcriptional corepressor results in aberrant mitochondrial function
Source: BMC Biochem. 2010 Jul 9;11:26. doi: 10.1186/1471-2091-11-26 (PMC2909972; doi:10.1186/1471-2091-11-26)
Supplement: Additional file 2 — sin3 mutant cells grow very poorly on solid agar plates containing non-fermentable carbon sources. This file contains images showing the growth of wild type and a sin3 mutant on solid agar plates containing YPD or non-fermentable carbon sources. [file 1471-2091-11-26-S2.PDF]

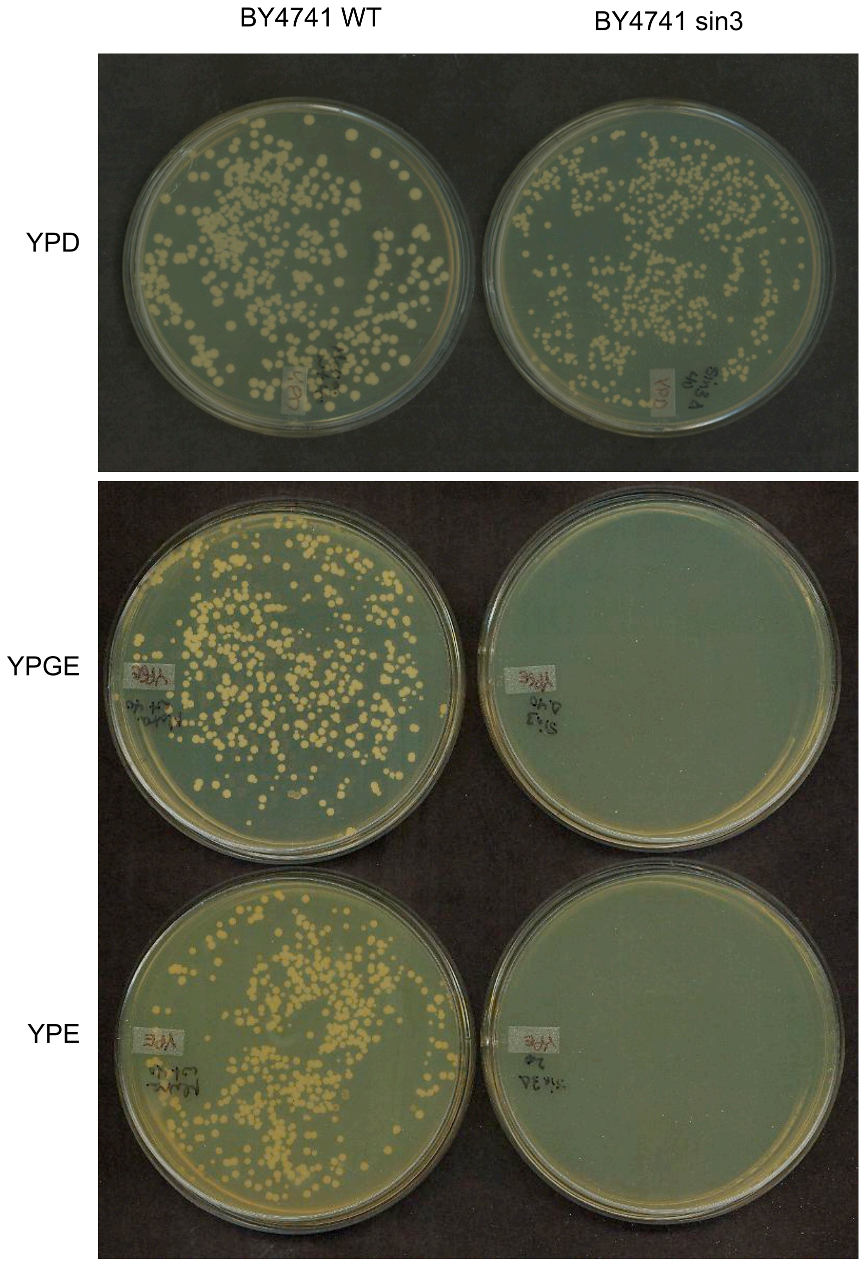

Additional file 2 - *sin3* mutant cells grow very poorly on solid agar plates containing non-fermentable carbon sources. Three hundred cells of wild type (BY4741 WT) and *sin3* null mutant (BY4741 sin3*)* were spread onto plates containing listed media. The top panel shows growth on YPD and the bottom panel shows growth on YPGE and YPE. YPD cultures were incubated at 30°C for 72 hours. YPE and YPGE cultures were incubated at 30°C for 144 hours.
