# Supplementary material for: Loss of the SIN3 transcriptional corepressor results in aberrant mitochondrial function
Source: BMC Biochem. 2010 Jul 9;11:26. doi: 10.1186/1471-2091-11-26 (PMC2909972; doi:10.1186/1471-2091-11-26)
Supplement: Additional file 3 — Additional S. cerevisiae strains demonstrate ySin3 is critical for growth in media prepared with non-fermentable carbon sources. This file shows growth curves for two additional S. cerevisiae strains, with differing genetic backgrounds, in media prepared with YPD or non-fermentable carbon sources. [file 1471-2091-11-26-S3.PDF]

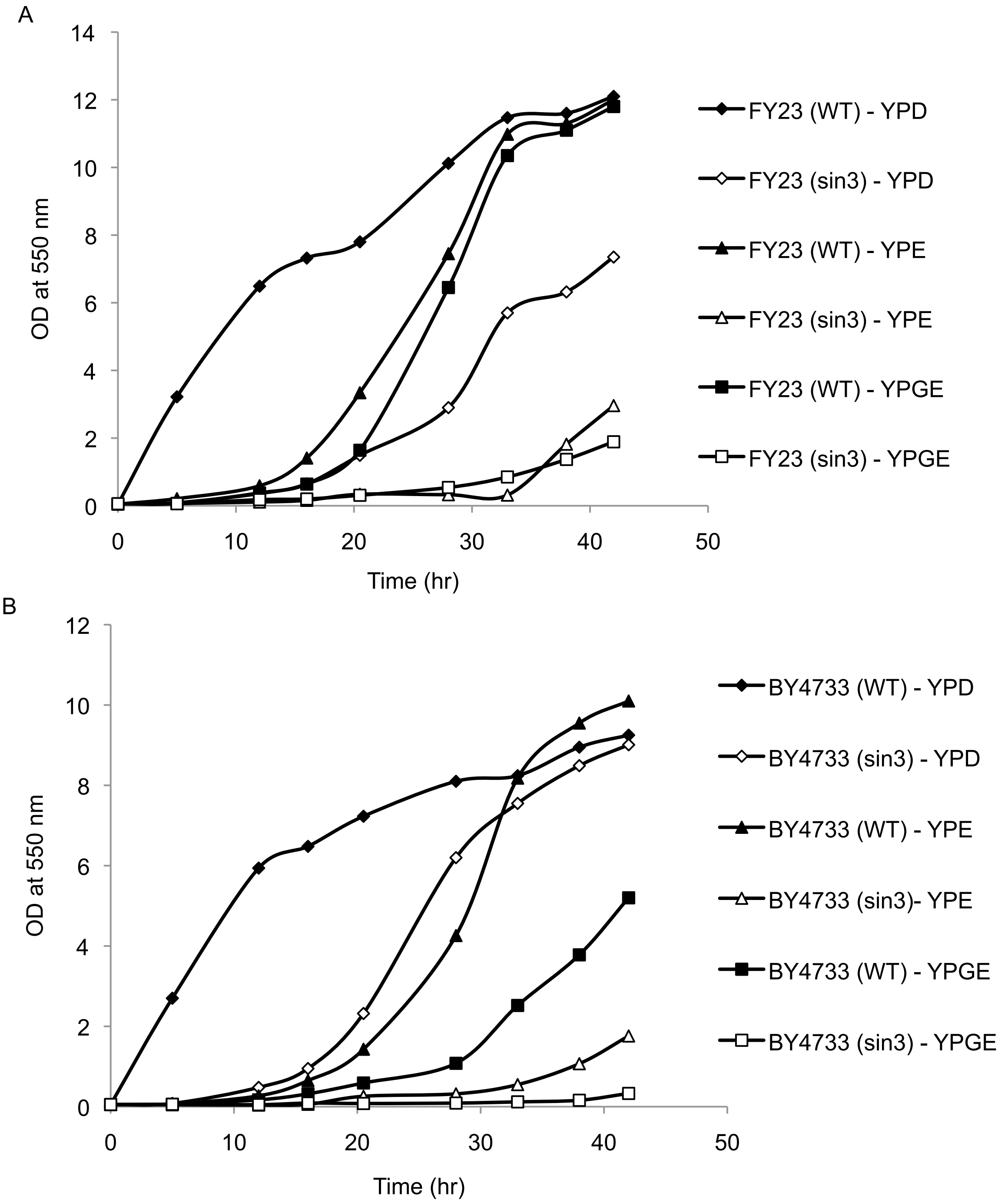


**Additional file 3 - Additional *S. cerevisiae* strains demonstrate ySin3 is critical for growth in media prepared with non-fermentable carbon sources.** Two independent *sin3* null mutants of differing genetic backgrounds were assayed for growth in liquid culture. Wild type (FY23 WT) and *sin3* null mutant (FY23 sin3) (A), wild type (BY4733 WT) and *sin3* null mutant (BY4733 sin3) (B) cells were grown in 5 ml of YPD, washed twice with water, inoculated into media containing glucose (YPD), ethanol (YPE) and glycerol and ethanol (YPGE) as sole carbon sources and incubated at 30°C. Absorbance at 550 nm was measured at the indicated times. Representative results of single growth curve assays are shown. Three independent trials were performed and all produced similar results.
