# Supplementary material for: Loss of the SIN3 transcriptional corepressor results in aberrant mitochondrial function
Source: BMC Biochem. 2010 Jul 9;11:26. doi: 10.1186/1471-2091-11-26 (PMC2909972; doi:10.1186/1471-2091-11-26)
Supplement: Additional file 4 — A second S. cerevisiae strain demonstrates ySin3 is critical for growth on media prepared with non-fermentable carbon sources. This file contains images showing the growth of wild type and a sin3 mutant of different genetic background on solid agar plates containing YPD or non-fermentable carbon sources. [file 1471-2091-11-26-S4.PDF]

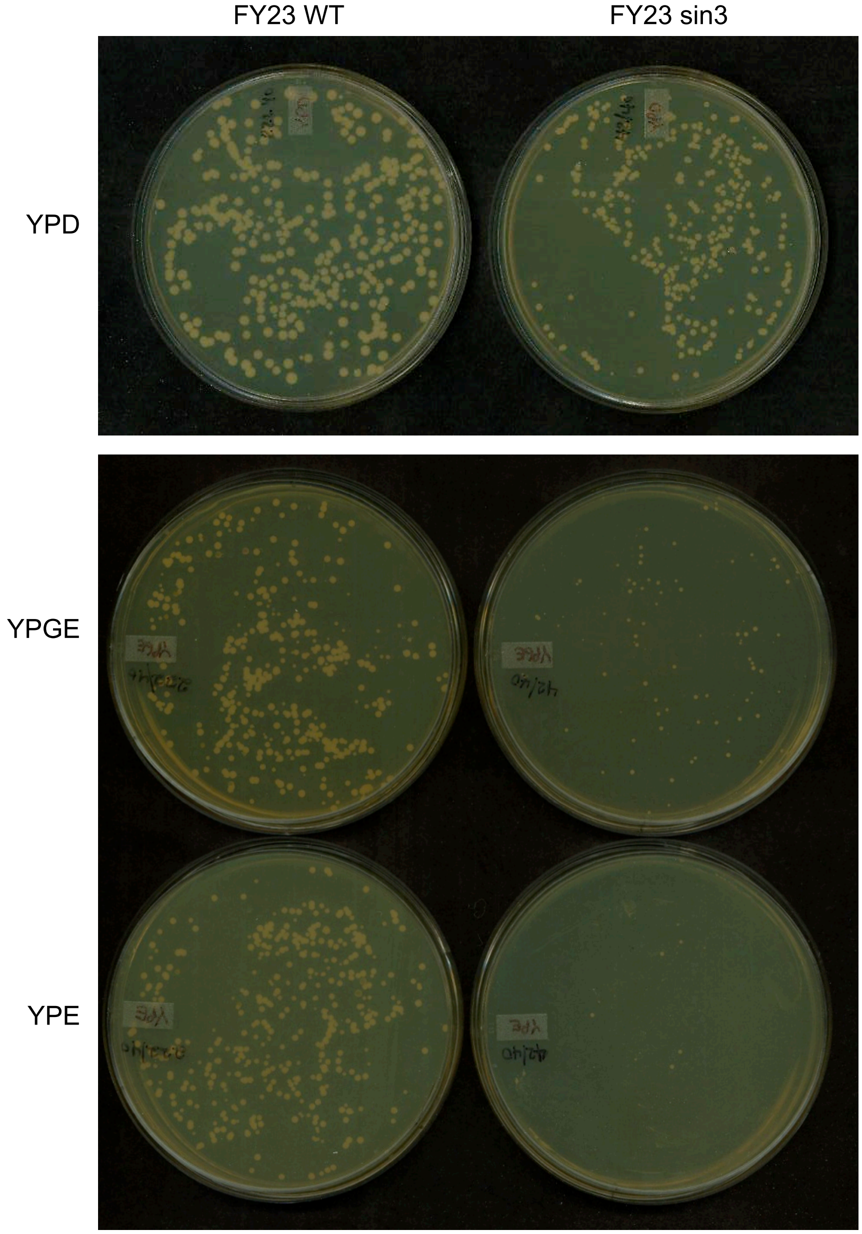

Additional file 4 - A second *S. cerevisiae* strain demonstrates ySin3 is critical for growth on media prepared with non-fermentable carbon sources. Three hundred cells of wild type (FY23 WT) and *sin3* null mutant (FY23 sin3) were spread onto plates containing the listed media. The top panel shows growth on YPD and the bottom panel shows growth on YPGE and YPE. YPD cultures were incubated at 30°C for 72 hours. YPE and YPGE cultures were incubated at 30°C for 144 hours.
