# Supplementary material for: Loss of the SIN3 transcriptional corepressor results in aberrant mitochondrial function
Source: BMC Biochem. 2010 Jul 9;11:26. doi: 10.1186/1471-2091-11-26 (PMC2909972; doi:10.1186/1471-2091-11-26)
Supplement: Additional file 6 — Mitochondrial genomes are observed in all sin3 and rpd3 mutants. This file contains images of DAPI staining of nuclear and mitochondrial DNA in wild type and sin3 and rpd3 mutant cells. [file 1471-2091-11-26-S6.PDF]

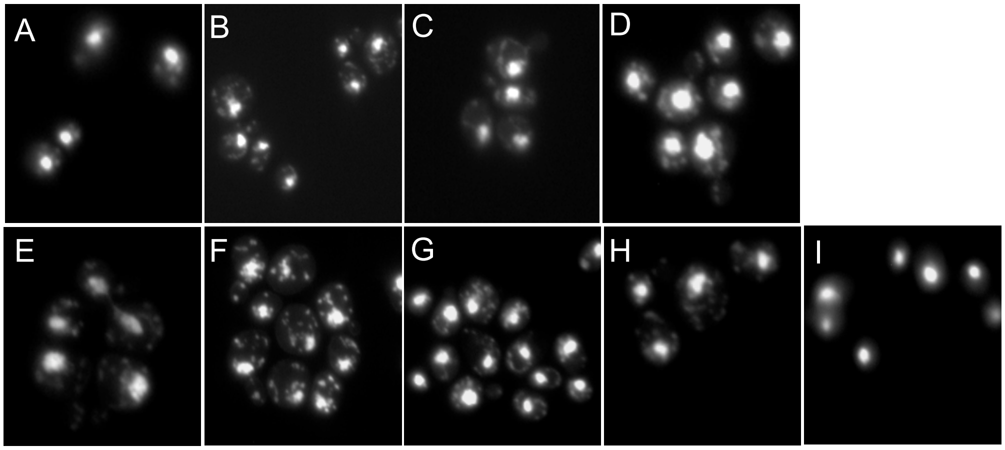

Additional file 6 - Mitochondrial genomes are observed in all *sin3* and *rpd3* mutants. DAPI staining of nuclear and mitochondrial DNA in the following strains: (A,E) Wild type (BY4741 WT), *sin3* null mutant (BY4741 sin3*), (B,F*) wild type (FY23 WT*), sin3* null mutant (FY23 sin3*), (C,G)* wild type (BY4733 WT*), sin3* null mutant (BY4733 sin3*), (D,H)* wild type (BY4742 WT*), and rpd3* null mutant (BY4742 rpd3) and (I) rho^{o} for comparison.
