# Supplementary material for: Loss of the SIN3 transcriptional corepressor results in aberrant mitochondrial function
Source: BMC Biochem. 2010 Jul 9;11:26. doi: 10.1186/1471-2091-11-26 (PMC2909972; doi:10.1186/1471-2091-11-26)
Supplement: Additional file 7 — rpd3 mutant cells grow very poorly on solid agar plates containing non-fermentable carbon sources. This file contains images showing the growth of wild type and an rpd3 mutant on solid agar plates containing YPD or non-fermentable carbon sources. [file 1471-2091-11-26-S7.PDF]

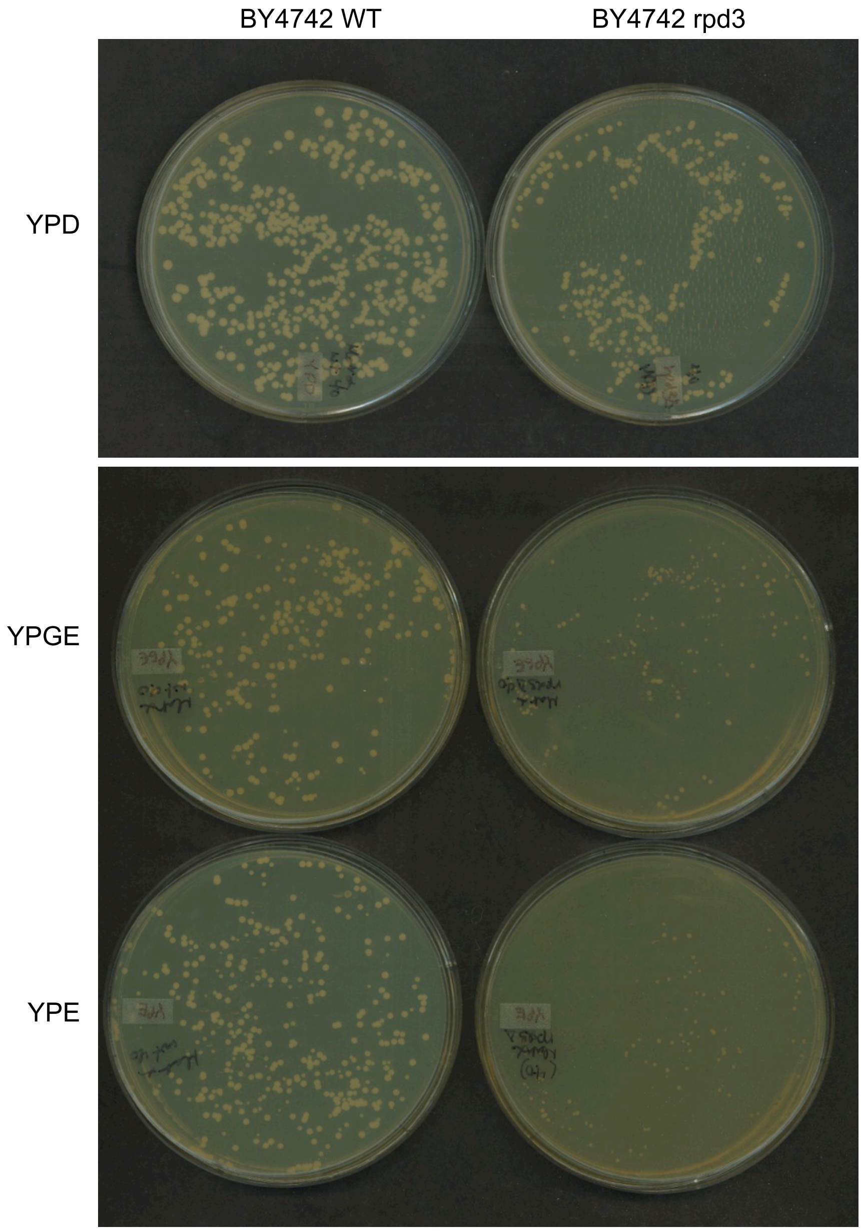

Additional file 7 - *rpd3* mutant cells grow very poorly on solid agar plates containing non-fermentable carbon sources. Three hundred cells of wild type (BY4742 WT) and *rpd3* null mutant (BY4742 rpd3) were spread onto plates containing listed media. The top panel shows growth on YPD and the bottom panel shows growth on YPGE and YPE. YPD cultures were incubated at 30°C for 72 hours. YPE and YPGE cultures were incubated at 30°C for 144 hours.
